# Analytical performance of a new automated chemiluminescent magnetic immunoassays for soluble PD-1, PD-L1, and CTLA-4 in human plasma

**DOI:** 10.1038/s41598-019-46548-3

**Published:** 2019-07-12

**Authors:** Megumi Goto, Kenji Chamoto, Keiko Higuchi, Saya Yamashita, Kenta Noda, Takuya Iino, Masahiro Miura, Toshinari Yamasaki, Osamu Ogawa, Makoto Sonobe, Hiroshi Date, Junzo Hamanishi, Masaki Mandai, Yoshimasa Tanaka, Shunsuke Chikuma, Ryusuke Hatae, Manabu Muto, Sachiko Minamiguchi, Nagahiro Minato, Tasuku Honjo

**Affiliations:** 1 Clinical Innovation, Sysmex Corporation, Hyogo, Japan; 2 Department of Immunology and Genomic Medicine, Graduate School of Medicine, Kyoto University, Kyoto, Japan; 3 Technology Development, Sysmex Corporation, Hyogo, Japan; 4 Central Research Laboratories, Sysmex Corporation, Hyogo, Japan; 5 Department of Urology, Graduate School of Medicine, Kyoto University, Kyoto, Japan; 6 Department of Thoracic Surgery, Graduate School of Medicine, Kyoto University, Kyoto, Japan; 7 Department of Gynecology and Obstetrics, Graduate School of Medicine, Kyoto University, Kyoto, Japan; 8 Center for Bioinformatics and Molecular Medicine, Graduate School of Biomedical Sciences, Nagasaki University, Nagasaki, Japan; 9 Department of Microbiology and Immunology, Keio University School of Medicine, Kyoto, Japan; 10 Department of Therapeutic Oncology, Graduate School of Medicine, Kyoto University, Kyoto, Japan; 11 Department of Pathology, Graduate School of Medicine, Kyoto University, Kyoto, Japan; 12 DSK Project, Medical Innovation Center, Graduate School of Medicine, Kyoto University, Kyoto, Japan; 13 Kyoto University Institute for Advanced Study, Kyoto, Japan

**Keywords:** Immune evasion, Tumour immunology, Biomarkers

## Abstract

Current clinically approved biomarkers for the PD-1 blockade cancer immunotherapy are based entirely on the properties of tumour cells. With increasing awareness of clinical responses, more precise biomarkers for the efficacy are required based on immune properties. In particular, expression levels of immune checkpoint-associated molecules such as PD-1, PD-L1, and CTLA-4 would be critical to evaluate the immune state of individuals. Although quantification of their soluble form leased from the membrane will provide quick evaluation of patients’ immune status, available methods such as enzyme-linked immunosorbent assays to measure these soluble factors have limitations in sensitivity and reproducibility for clinical use. To overcome these problems, we developed a rapid and sensitive immunoassay system based on chemiluminescent magnetic technology. The system is fully automated, providing high reproducibility. Application of this system to plasma of patients with several types of tumours demonstrated that soluble PD-1, PD-L1, and CTLA-4 levels were increased compared to those of healthy controls and varied among tumour types. The sensitivity and detection range were sufficient for evaluating plasma concentrations before and after the surgical ablation of cancers. Therefore, our newly developed system shows potential for accurate detection of soluble PD-1, PD-L1, and CTLA-4 levels in the clinical practice.

## Introduction

Immune checkpoint molecules are one of the most important regulators in the immune system that maintain homeostasis by controlling immune reactions^1–3^. Among the family of immune checkpoints, cytotoxic T-lymphocyte-associated antigen 4 (CTLA-4) and programmed cell death-1 (PD-1) are the main players inhibiting the activation of T cell-mediated immune responses^3^. Thus, drugs regulating CTLA-4 and PD-1 function, especially blocking antibodies, are currently available and widely used for cancer treatment worldwide^4^. However, not all patients will benefit from this treatment; thus, identification of biomarkers to predict responders and non-responders, and/or potential side effects such as autoimmune-related diseases is urgently required.

Originally, PD-1 was discovered as a T cell apoptosis-associated molecule^5^. However, the observation that PD-1-deficient mice developed autoimmune diseases led to the conclusion that PD-1 is a negative regulator of immune responses^6^. One of the ligand of PD-1, programmed cell death-1 ligand-1 (PD-L1) is ubiquitously expressed throughout several tissues of the body, whereas PD-1 is mainly expressed on activated lymphocytes. When PD-1 binds to PD-L1, the PD-1 signal suppresses activated T cells through the recruitment of SHP-2, which dephosphorylates and inactivates Zap70, a major integrator of antigen receptor-mediated signals^7,8^. PD-L1 is aberrantly expressed on macrophage-lineage cells and tumour cells, resulting in the strong suppression of tumour-reactive T cells.

CTLA-4 is a member of the immunoglobulin superfamily and is highly homologous to another T cell surface receptor, CD28 which is critical for T cell activation^9,10^. Both CD28 and CTLA-4 bind to CD80 and CD86 on antigen-presenting cells. However, CTLA-4 binds to CD80 and CD86 with greater affinity and avidity than CD28, thus outcompeting CD28 and inhibiting T cell activation. Thus, CTLA-4 is critical for self-tolerance, attenuating autoimmunity and anti-tumour immunity^11,12^.

Numerous surface molecules assume two forms of expression: cell membrane-bound protein and a soluble form that is usually generated by proteolytic cleavage^13^. Splice variants of PD-1 and CTLA-4, or cleaved PD-1, PD-L1, and CTLA-4, have been identified as soluble forms in the blood^14–17^. Given the extensive involvement of these immune-regulating molecules in immune reactions and immune-related diseases, detailed investigations of the soluble forms of PD-1, PD-L1, and CTLA-4 can offer insight into new biomarker targets. In particular, high serum concentrations of the soluble PD-1 (sPD-1), PD-L1 (sPD-L1), and CTLA-4 (sCTLA-4) have been associated with several autoimmune diseases (e.g., Graves’ disease, myasthenia gravis, systemic lupus erythematosus, type 1 diabetes, systemic sclerosis, coeliac disease, autoimmune pancreatitis, and primary biliary cirrhosis)^18–20^.

Although the levels of sPD-1, sPD-L1, and sCTLA-4 in serum and/or plasma could serve as biomarkers of immune-related diseases, several technological issues have limited their clinical application. For instance, enzyme-linked immunosorbent assay (ELISA) is typically used to measure the levels of sPD-1, sPD-L1, and sCTLA-4, but is known to suffer from poor precision and reproducibility due to the manual procedures of experiments^21,22^. To overcome these limitations, we developed an automated measurement system for sPD-1, sPD-L1, and sCTLA-4 based on a chemiluminescent enzyme immunoassay (HISCL system). Overall, our developed method can offer a new tool for the accurate measurement of sPD-1, sPD-L1, and sCTLA-4 in clinical settings, and allow for further identification of biomarkers of immune responses.

## Results

### Standard curve

**Figure 1:**
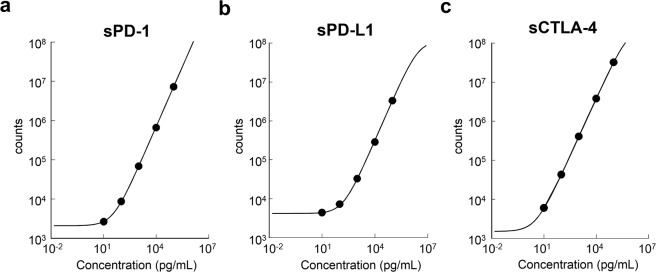
Relationship between relative photon counts and sPD-1 (**a**), sPD-L1 (**b**), and sCTLA-4 (**c**) levels. Serial dilutions of His-tagged PD-1, PD-L1, and CTLA-4 were analyzed and fit into a four-parameter logistic regression model.

We chose the best combination of two antibodies for PD-1, PD-L1 and CTLA-4 to obtain the highest intensity of chemiluminescent values using HISCL as described in Materials and Methods. These sPD-1, sPD-L1, and sCTLA-4 detection assays were standardized using recombinant human PD-1, PD-L1, and CTLA-4. Chemiluminescent signals are reportable as relative light units and directly correlate with the amount of PD-1, PD-L1, and CTLA-4. Concentrations of PD-1, PD-L1 and CTLA-4 were then determined by interpolation from an unweighted four-parameter standard curve obtained by serially diluted calibrators of the recombinant proteins (Fig. 1).

### Sensitivity

The limit of blanks (LoBs) in these assays for sPD-1, sPD-L1, and sCTLA-4 detection were calculated with the measurements of 60 zero standards. The LoBs for sPD-1, sPD-L1, and sCTLA-4 were 1.04 pg/mL, 0.00 pg/mL, and 0.11 pg/mL, respectively. The limit of detections (LoDs) and limit of quantitations (LoQs) were tested using five samples with different concentrations. LoDs of sPD-1, PD-L1, and sCTLA-4 were 3.92 pg/mL, 6.79 pg/mL, and 0.39 pg/mL; and LoQs were 10.0 pg/mL, 25.0 pg/mL, and 1.0 pg/mL, respectively, based on the criterion of a 20% coefficient of variation (CV).

### Reproducibility

Control samples with high, mid, and low concentrations were used to evaluate assays for reproducibility during the validation runs. The range of CV (%) from the samples prepared from low to high concentrations for sPD-1, sPD-L1, and sCTLA-4 assays was <2.7%, 3.7–5.8%, and 2.0–10.9%, respectively (Table 1).

**Table 1:** Twenty-day assay precision evaluation.

Level^a^		sPD-1			sPD-L1			sCTLA-4		
		L	M	H	L	M	H	L	M	H
n (Individual)		120	120	120	120	120	120	120	120	120
Mean (pg/mL)		164.8	1003.8	4628.3	210.4	1098.7	5237.4	1.2	1032.1	5574.2
Within-day	CV(%)	2.5	3.0	3.3	5.8	2.3	1.6	12.6	3.5	4.4
	SD	4.8	36.7	185.9	13.3	21.9	65.8	0.1	37.4	218.2
Between-day	CV(%)	2.1	2.3	1.9	4.1	2.5	2.0	3.2	1.7	1.9
	SD	3.5	22.8	87.9	8.6	26.9	104.1	0.1	17.7	103.7
Overall	CV(%)	2.7	2.7	2.7	5.8	3.7	3.7	10.9	3.5	2.0
	SD	4.5	26.9	124.3	12.2	41.2	194.1	0.1	36.4	10.9

^a^L, M, and H indicate low, middle, and high concentration controls, respectively.

SD, standard deviation; CV, coefficient of variation.

### Interference

Interference of common blood components was assessed by adding interfering substances into plasma samples. As shown in Table 2, the changes between samples with and without typical interfering substances in plasma were minor or negligible in the physiological concentration (less than 5% change in sPD-1, sPD-L1, and sCTLA-4 concentrations).

**Table 2:** Interference testing with chyle, hemoglobin, bilirubin, rheumatoid factor, and EDTA- 2Na.

Interfering substances	Concentrations	Mean % difference in concentration		
		sPD-1	sPD-L1	sCTLA-4
Chyle	1660 FTU	−1.2	1.3	−2.6
Hemoglobin	4900 mg/L	1.4	1.8	−0.6
Free bilirubin	200 mg/L	1.5	−1.4	−3.1
Ditauro-bilirubin	200 mg/L	0.0	0.0	−0.4
Rheumatoid factor	500 IU/mL	−1.4	1.4	−1.3
EDTA-2Na	5000 ug/mL	−0.2	0.3	−3.9

### Dilution linearity

**Figure 2:**
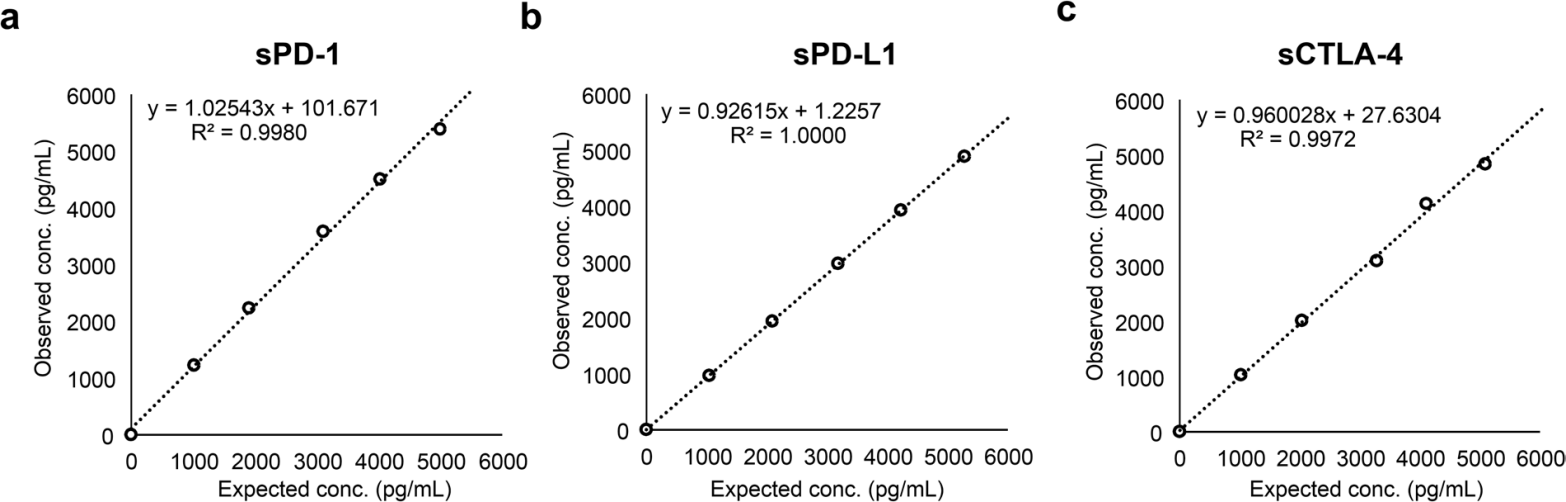
Dilution linearity of HISCL sPD-1 (**a**), sPD-L1 (**b**), and sCTLA-4 (**c**) assays using serially diluted samples. Human plasma spiked with recombinant PD-1/PD-L1/CTLA-4 was diluted with HISCL dilution buffer.

The dilution linearity of this immune assay system was assessed by measuring the five points with different concentrations obtained by diluting the same specimens five times. Specimen dilution analysis yielded linear results across the dynamic range of the assay. The average correlation between observed and expected values of sPD-1, sPD-L1, and sCTLA-4 were >0.99. The sPD-1, sPD-L1, and sCTLA-4 assays were all found to be linear between 0.0 and 5277.4, 5289.1, and 5090.8 pg/mL, respectively. The linear relationships with slopes corresponded to 1.025 ± 0.0, 0.926 ± 0.0, and 0.960 ± 0.02, respectively (Fig. 2).

### Spike recovery

The mean (range) recovery of sPD-1, sPD-L1, and sCTLA-4 calculated by spiking recombinant PD-1, PD-L1, and CTLA-4 protein into ten endogenous plasma specimens of healthy donors were 107% (102–114%), 103% (96–117%), and 98% (92–104%) for 100 pg/mL (L in Table 3); 108% (101–112%), 94% (84–109%), and 98% (92–104%) for 250 pg/mL (M in Table 3); and 107% (101–111%), 98% (95–107%), and 100% (93–105%) for 1000 pg/mL (H in Table 3).

**Table 3:** Spike recovery testing.

Spike recovery	sPD-1			sPD-L1			sCTLA-4		
Level^a^	L	M	H	L	M	H	L	M	H
n (Individual)	10	10	10	10	10	10	10	10	10
spike conc. (pg/mL)	100.7	248.2	962.2	89.8	235.6	938.9	96.2	237.1	949.5
recovery (%)	107.1	108.0	107.9	103.2	93.9	98.1	98.5	98.3	100.3

Ten normal human plasma specimens were spiked at three levels of concentration with recombinant PD-1, PD-L1 and CTLA-4.

^a^L, M, and H indicate low, middle, and high concentration controls, respectively.

### Specificity

**Figure 3:**
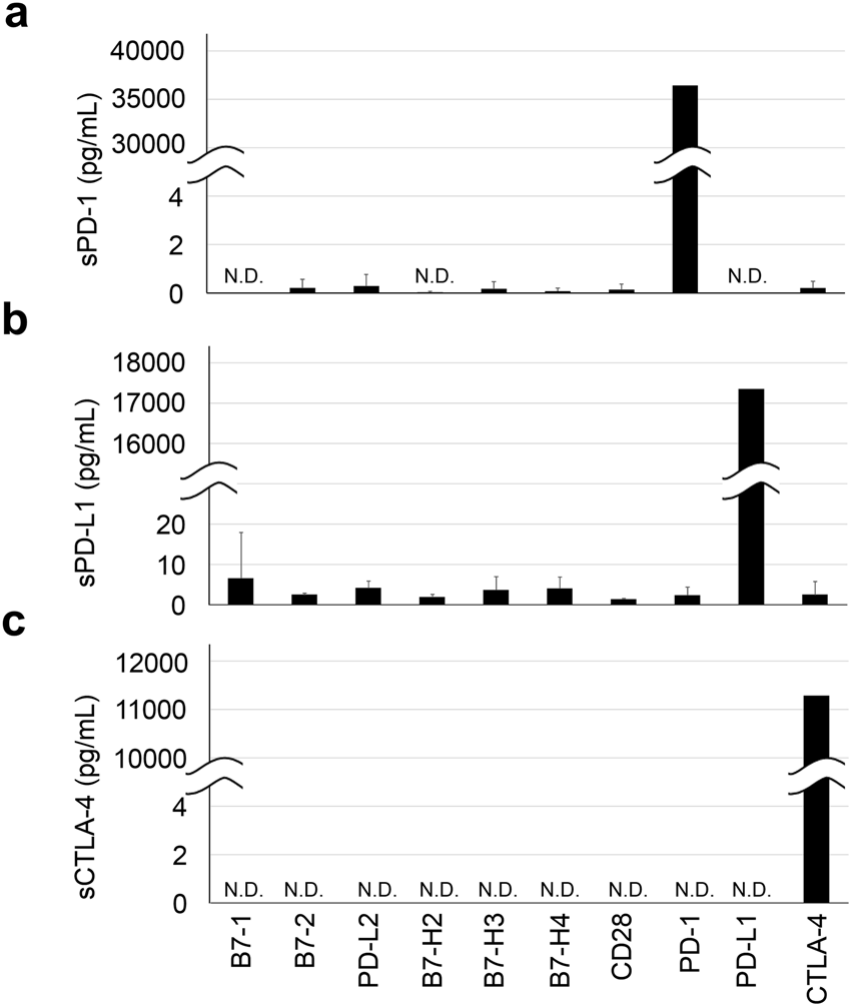
Specificity of HISCL sPD-1, sPD-L1, and sCTLA-4 immunoassays. PD-1, PD-L1, CTLA-4, and other immune checkpoint molecules (B7-1, B7-2, PD-L2, B7-H2, B7-H3, B7-H4, and CD28) were diluted to 10 nM each and detected by the HISCL PD-1 (**a**), PD-L1 (**b**), and CTLA-4 (**c**) immunoassays.

To evaluate the specificity of the assay system, other soluble homologous proteins in the same superfamily (PD-1, PD-L1, CTLA-4, B7-1, B7-2, B7-H2, B7-H3, B7-H4, CD28, and PD-L2) were measured and compared with the values of sPD-1, sPD-L1, and sCTLA-4. Our assay system detected sPD-1, sPD-L1, and sCTLA-4 with less than 0.05% differences when the same molecular concentration of other homologous proteins was measured (Fig. 3).

### Application of the automated HISCL system to the detection of plasma sPD-1, sPD-L1, and sCTLA-4 in cancer patients and healthy controls

**Figure 4:**
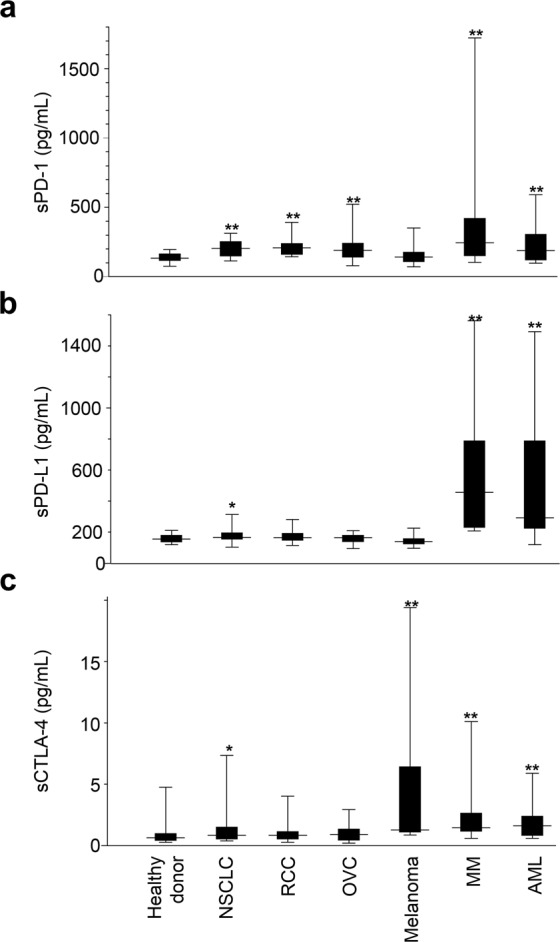
Levels of sPD-1 (**a**), sPD-L1 (**b**), and sCTLA-4 (**c**) in plasma samples from healthy donors and tumour patients. Healthy, n = 50; NSCLC patients, n = 35; RCC patients, n = 33; OVC patients, n = 17; melanoma patients, n = 15; MM patients, n = 18; AML patients, n = 17. The Mann-Whitney U test was used for comparisons between healthy donor and each tumor patients. One asterisk (*) indicates *p*-value smaller than 0.05 (*p* < 0.05). Two asterisks (**) indicate *p*-value smaller than 0.01 (*p* < 0.01).

As shown in Fig. 4, the sPD-1 level was found to be significantly higher in plasma samples from patients with non-small cell lung cancer (NSCLC), renal cell carcinoma (RCC), ovarian cancer (OVC), Multiple Myeloma (MM), and acute myeloid leukaemia (AML) than that detected in healthy donors. The sPD-L1 level was significantly higher in samples from patients with NSCLC, MM, and AML than that in healthy donors. Similarly, the sCTLA-4 level was significantly higher in plasma samples from patients with NSCLC, melanoma, MM, and AML than in samples from healthy donors. These data indicate that sPD-1, sPD-L1, and sCTLA-4 levels in plasma differ according to the tumour type, and confirm the elevated immune reactions in cancer patients.

### Tumour type-dependent regulation of plasma sPD-1, sPD-L1, and sCTLA-4 levels

**Figure 5:**
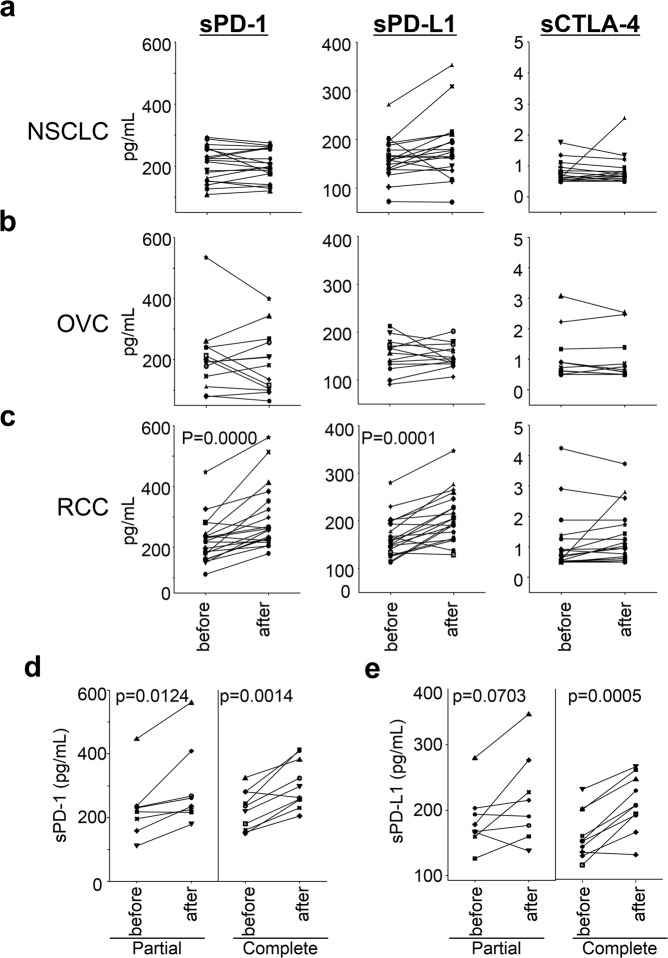
Concentrations of sPD-1, sPD-L1, and sCTLA-4 in plasma from NSCLC (**a**), OVC (**b**), and RCC (**c**) patients before/after surgery. Levels of sPD-1, sPD-L1, and sCTLA-4 were measured from the patients prior to undergoing surgery and at the 4-week post-surgical visit to the hospital. Concentrations of sPD-1 (**d**) and sPD-L1 (**e**) in plasma from RCC patients that received partial or complete nephrectomy, respectively. Differences between before and after the surgery groups were assessed by Student t-test.

In contrast to our expectation, there were no significant differences in the plasma levels of sPD-1, sPD-L1, and sCTLA-4 before and after the surgery in NSCLC and OVC patients; however, significant differences in sPD-1 and sPD-L1 levels were detected in RCC patients (Fig. 5a–c). Moreover, compared partial with complete in RCC patients that received nephrectomy, the results showed a greater difference in sPD-1 and sPD-L1 levels at before and after surgery (Fig. 5d,e).

## Discussion

Currently approved predictive biomarkers for the clinical responses to PD-1 blockade cancer immunotherapy are PD-L1 expression on tumours and the tumour mutation burden^23,24^. However, these tumour-derived biomarkers are not very reliable for accurate prediction. Since the immune response is controlled by the properties of both the tumour and the immune system, biomarkers that reflect immune properties are needed. Although PD-1, PD-L1, and CTLA-4 are known as critical immune checkpoint-associated molecules, measuring their expression levels in each tissue are quite difficult. Thus, it would be ideal to reliably measure sPD-1, sPD-L1, and sCTLA-4 levels in the blood, since the soluble forms of these three molecules are known to be cleaved from the cell surface and reflect total expression levels in the body. However, available detection systems for sPD-1, sPD-L1, and sCTLA-4 are relatively immature. Thus, we used the automated immunochemistry analyser HISCL, which is based on chemiluminescent magnetic immunoassay technology, to develop a rapid method for the detection of sPD-1, sPD-L1, and sCTLA-4 with good precision, reproducibility, and high sensitivity.

HISCL is widely used for *in vitro* diagnostics (IVD) in several diseases such as hepatitis, infectious diseases, cardiac disease, cancer, sepsis, and atopic dermatitis^25–29^. The main advantages of HISCL over ELISA are as follows: (1) due to the liquid-liquid reaction base at 42 °C of the highly-reactive substrate, the reaction is very rapid (17 min) and highly sensitive; (2) the larger amounts of antibodies and substrates in liquids provide more capacity for the reaction, expanding the dynamic range from the pico- to milligram scale; and (3) all processes are fully automated, resulting in high reproducibility. These three conditions are essential for clinical use, especially considering the necessity of sharing cut-off values between different facilities.

Since tumour tissues are the main site of the immune responses, activated T cells expressing PD-1 and CTLA-4 are trafficked into tumour tissues. Thus, PD-L1 expression in tumour cells is one of the important predictive biomarkers for clinical responses in the PD-1 blockade cancer immunotherapy for NSCLC. In this context, we compared the plasma concentrations of sPD-1, sPD-L1, and sCTLA-4 between healthy donors and tumour bearing patients. It is demonstrated that the plasma concentrations of sPD-1, sPD-L1, and/or sCTLA-4 were higher than those of healthy donors, with some differences according to tumour types, implying that anti-tumour immunity has launched in tumour bearing patients. Although sPD-1 is mainly derived from T and B cells, sCTLA-4 exclusively from T cells, and sPD-L1 from various organs^3^, it is difficult to define the specific cells in each organ that release these soluble factors. To investigate the impact of cancer tissues for the plasma levels of the three markers, we compared them before and after the surgical removal of tumour tissues in patients with early stage of NSCLC, OVC, and RCC. Our results confirmed that the concentrations of sPD-1, sPD-L1, and sCTLA-4 are likely more associated with immune reactions probably in the lymphoid organs rather than in the tumour tissues of patients, as there were no differences in these levels before and after surgery except RCC patients. In RCC patients, the increment rate of sPD-1 and sPD-L1 after complete removal of the kidney were greater than that observed after partial removal, suggesting that sPD-1 and sPD-L1 can be partially eliminated by the kidney. Thus, it is likely that the kidney is one of the main sites for the regulation of sPD-1 and sPD-L1 levels in the blood.

In summary, we established rapid and sensitive assay systems for sPD-1, sPD-L1, and sCTLA-4 with a wide detection rage. The systems are fully automated, thereby providing high reproducibility. The important point for clinical application is determination of cut off values. Therefore, obtaining absolute values without variation between different facilities is critical for biomarker studies. Our established method meets this requirement. We have planned clinical studies to stratify the cancer patients treated by immune checkpoint inhibitors  based on these biomaekers. Considering the importance of immune checkpoints and accumulating evidence of cancer immunotherapy, our novel systems can provide new opportunities to identify biomarkers of cancer immunotherapy and other immune-related diseases. This method would be useful to stratify the patients for “personalized” medicine.

## Materials and Methods

### Human samples

Plasma in di-sodium/-potassium dihydrogen ethylenediaminetetraacetic acid (EDTA) tubes from healthy donors or patients with different tumour types [NSCLC (stage III and IV), RCC (stage III, IV and recurrent), OVC (stage II–IV), melanoma, MM, and AML] were purchased from ProMedDx, LCC (Norton, MA, USA). We further assessed the changes of sPD-1, sPD-L1, and sCTLA-4 concentrations in the plasma of cancer patients [NSCLC (stage I, II), RCC (stage I, III), and OVC (stage I–IV)] enrolled from our institute. All subjects signed an informed consent form, and all protocols were approved by the Kyoto University Institutional Review Board. All methods were performed in accordance with the relevant guidelines and regulations.

### Assay description

Antibodies using assay system were conjugated. Anti-PD-1 antibody (MIH4, Thermo Fisher Scientific MA, USA), anti-PD-L1 antibody (27A2)^30^ and anti-CTLA-4 antibody (14D3, Thermo Fisher Scientific) were biotinylated. And anti-PD-1 antibody (PD1.3.1.3, Miltenyi Biotec, Bergisch Gladbach, Germany), anti-PD-L1 antibody (9L814, US Biological, MA, USA), and anti-CTLA-4 antibody (BNI3, TONBO Biosciences, CA, USA) were conjugated to alkaline phosphatase.

sPD-1, sPD-L1, and sCTLA-4 detection assays were standardized using commercially available purified recombinant human PD-1, PD-L1, and CTLA-4 with a C-terminal His-tag (Sino Biological Inc., Beijing, China).

A two-step monoclonal antibody sandwich system was used to quantitatively determine the concentrations of sPD-1, sPD-L1, and sCTLA-4 in human plasma using immunoassay reagents developed based on the HISCL-800 system (Sysmex, Hyogo, Japan). In the HISCL system, soluble immune checkpoint molecules were captured by each specific biotinylated antibody, and the bound products were incubated with alkaline phosphatase (ALP) conjugated antibodies (ALP-anti-PD-1, ALP-anti-PD-L1, ALP-anti-CTLA-4). Two reagent packs (sPD-1, sPD-L1, and sCTLA-4 detection pack and a chemiluminescence substrate pack) were loaded in the HISCL-800 system. The detection pack comprised three reagents: a capture antibody buffer solution (R1), a streptavidin magnetic beads solution (R2), and an ALP-antibody solution (R3). The chemiluminescence substrate reagent pack contained a dilution buffer solution (R4) and CDP-Star substrate solution (R5).

Plasma samples (20 μL) were diluted to 50 μL with R1 and then mixed with R2 (30 μL). After the binding reaction, bound/free (B/F) separation was performed. R3 (100 μL) was added to the obtained sample and mixed well. Subsequently, after B/F separation, and mixed well with R4 (50 μL) and R5 (100 μL) before reading the fluorescence signal. The chemiluminescent intensity was acquired within a period of 17 min following the operation described above. The reaction chamber was maintained at 42 °C throughout the procedure.

### Sensitivity

The LoB, LoD, and LoQ values were calculated according to the Clinical Laboratory Standards Institute (CLSI) EP17-A2 guideline^31^. The LoB was analysed using a zero calibrator matrix. The LoD and LoQ values were evaluated using five points with different concentrations using recombinant human PD-1, PD-L1, and CTLA-4 diluted with calibration buffer. Values were determined by triplicate samples twice with two different reagent lots. Measurements were repeated three times on three different days (n = 18 for each point).

### Reproducibility

Assay precision was evaluated according to the CLSI EP05-A3 guideline following a 20 × 2 × 2 experimental design^32^. We pooled normal human plasma samples as a low-concentration control. Subsequently, middle- and high-concentration panels were prepared by adding recombinant proteins to the pooled plasma. The panels were run in replicates of six, twice daily with a 2 hours or greater gap between runs. The human plasma panel represents the native endogenous sPD-1, sPD-L1, and sCTLA-4 concentrations (for low-concentration group). For the middle- and high-concentration groups, recombinant human PD-1, PD-L1, and CTLA-4 were spiked into the low-concentration control up to 1188 pg/mL, 538 pg/mL, and 877 pg/mL, respectively. For the high-concentration panel, recombinant PD-1, PD-L1, and CTLA-4 proteins were added up to 5455 pg/mL, 2654 pg/mL, and 4789 pg/mL, respectively.

### Interfering substances

Potential interference materials were added to the plasma pool of healthy donors up to the following concentrations: chyle (0–1660 FTU), haemoglobin (0–4900 mg/L), free-form bilirubin (0–200 mg/L), conjugated-form bilirubin (0–200 mg/L), rheumatoid factor (RF; 0–500 IU/mL), and EDTA-2Na (0–5000 μg/mL). The free-form and conjugated-forms of bilirubin, haemoglobin, lipids, and RF were obtained from interference Check A Plus and Interference Check RF (Sysmex, Hyogo, Japan).

### Dilution linearity

Dilution linearity was assessed according to CLSI guideline EP06-A^33^. Samples of human plasma were spiked with recombinant PD-1, PD-L1, and CTLA-4 and then diluted five times with HISCL dilution buffer.

### Specificity

The specificity of assay system was also determined by other homologous proteins in the same superfamily. PD-1, PD-L1, CTLA-4, B7-1, B7-2, B7-H2, B7-H3, B7-H4, CD28, and PD-L2 with a C-terminal His-tag were purchased from Sino Biological. These proteins were prepared 10 nM concentration with calibration buffer and measured triplicate by our assay system.

### Statistical analysis

Statistical analysis was performed using StatFlex v.6.0 software (Artech Co. Ltd., Osaka, Japan) and was expressed as Mean, Standard Deviation (SD) and Coefficient of Variation (CV) for each test parameter. Linearity was assessed by linear regression analysis. The Mann-Whitney U test was used for comparisons between healthy donor and each tumour patients. Differences between before and after the surgery groups were assessed by Student t-test, and a *P*-value < 0.05 was considered to be statistically significant.

## Data Availability

The datasets generated during and/or analyzed during the current study are available from the corresponding author on reasonable request.
